# Lesion Recognition and Cleavage of Damage-Containing Quadruplexes and Bulged Structures by DNA Glycosylases

**DOI:** 10.3389/fcell.2020.595687

**Published:** 2020-11-30

**Authors:** Alexandra A. Kuznetsova, Olga S. Fedorova, Nikita A. Kuznetsov

**Affiliations:** ^1^Institute of Chemical Biology and Fundamental Medicine of SB RAS, Novosibirsk, Russia; ^2^Department of Natural Sciences, Novosibirsk State University, Novosibirsk, Russia

**Keywords:** base excision repair, DNA glycosylase, G-quadruplex, DNA bulge, pre-steady-state kinetics, fluorescence

## Abstract

Human telomeres as well as more than 40% of human genes near the promoter regions have been found to contain the sequence that may form a G-quadruplex structure. Other non-canonical DNA structures comprising bulges, hairpins, or bubbles may have a functionally important role during transcription, replication, or recombination. The guanine-rich regions of DNA are hotspots of oxidation that forms 7,8-dihydro-8-oxoguanine, thymine glycol, and abasic sites: the lesions that are handled by the base excision repair pathway. Nonetheless, the features of DNA repair processes in non-canonical DNA structures are still poorly understood. Therefore, in this work, a comparative analysis of the efficiency of the removal of a damaged nucleotide from various G-quadruplexes and bulged structures was performed using endonuclease VIII-like 1 (NEIL1), human 8-oxoguanine-DNA glycosylase (OGG1), endonuclease III (NTH1), and prokaryotic formamidopyrimidine-DNA glycosylase (Fpg), and endonuclease VIII (Nei). All the tested enzymes were able to cleave damage-containing bulged DNA structures, indicating their important role in the repair process when single-stranded DNA and intermediate non–B-form structures such as bubbles and bulges are formed. Nevertheless, our results suggest that the ability to cleave damaged quadruplexes is an intrinsic feature of members of the H2tH structural family, suggesting that these enzymes can participate in the modulation of processes controlled by the formation of quadruplex structures in genomic DNA.

## Introduction

Exogenous and endogenous agents such as very reactive cell metabolites, external environmental compounds, and ionizing or UV irradiation constantly damage cellular DNA. The main sources of endogenous damage to DNA are reactive oxygen species (ROS). The latter give rise to various DNA base lesions, including thymine glycol (Tg), 7,8-dihydro-8-oxoguanine (8-oxoguanine, oxoG), 4,6-diamino-5-formamidopyrimidine (Fapy-A), and 2,6-diamino-4-hydroxy-5-formamidopyrimidine (Fapy-G; Wallace, 2002; Evans et al., 2004). Damages to DNA bases can be mutagenic and when unrepaired may result in blockage of DNA polymerases, base mispairing, and finally genomic instability (Wallace, 2002). The bulk of this endogenous burden is managed by base excision DNA repair (BER). The latter involves a few DNA *N*-glycosylases specific to the removal of a large spectrum of alkylated, oxidized, or deaminated bases, and sometimes normal mispaired bases; this process generates abasic (apurinic/apyrimidinic; AP) sites in DNA (Friedman and Stivers, 2010; Svilar et al., 2011).

Aside from the canonical duplex structure, DNA can populate a large variety of states, from four-stranded arrangements to single-stranded conformations. Guanine-rich nucleic acid molecules can fold into non-B form RNA or DNA structures called G-quadruplexes. These are secondary structures formed by four strands of certain G-rich sequences of nucleic acids. They are a consequence of stacking of multiple stable “G-quartets,” i.e., planar arrangements of four guanines held together by Hoogsteen hydrogen bonds and additionally stabilized by monovalent cations (usually Na^+^ or K^+^; Sen and Gilbert, 1988, 1990). G-quadruplex topologies can be categorized into several types, such as parallel, antiparallel, and hybrid (Wang and Patel, 1993; Parkinson et al., 2002; Ambrus et al., 2006; Phan et al., 2006, 2007; Dai et al., 2007). Typically, the syn/anti-glycosidic conformation of guanines is regarded as a major factor for the folding of the G-quadruplex structure. There is specific nomenclature for G4 folds in the repeat sequence of the telomere, where the parallel fold is termed the propeller, and the antiparallel fold coordinately bound to potassium ions is termed the hybrid and can have two conformations (Xu, 2011). In the parallel folds, loops are double-strand reversals, and in antiparallel folds, loops are edgewise, diagonal, and/or double-strand reversals.

The structure and function of G-quadruplexes have aroused much interest. Recently, they were implicated in some human genetic neurodegenerative diseases (Haeusler et al., 2014; Brčić and Plavec, 2015). G-quadruplexes are thought to be also crucial for DNA replication (Besnard et al., 2012; Valton et al., 2014), telomere maintenance (Paeschke et al., 2005, 2008; Xu, 2011), genome rearrangements (Ribeyre et al., 2009; Lemmens et al., 2015), the DNA damage response (Rodriguez et al., 2012; Lopez et al., 2017), chromatin structure (Hänsel-Hertsch et al., 2016; Mao et al., 2018), RNA processing (Kwok et al., 2016), and transcriptional (Fernando et al., 2009; David et al., 2016), or translational regulation (Kumari et al., 2007; Kwok et al., 2015). Human telomeres consist of a 100–200 bp 3’ single-stranded overhang with 5’-TTAGGG-3’ repeats (McElligott and Wellinger, 1997).

It should be mentioned that the most important oxidative damaged bases in the 5’-TTAGGG-3’ context are Tg and oxoG: the major oxidation products of DNA thymine and guanine, respectively. It is known that Tg is an effective block for DNA polymerases and consequently a lesion lethal to the cell, whereas oxoG is a premutagenic lesion causing a mispairing with adenine (Wallace, 2002; Fleming and Burrows, 2017).

It has been shown previously that guanine-to-8-oxoguanine (Vorlícková et al., 2012b) or guanine-to-adenine (Tomaško et al., 2009) substitutions and guanine abasic lesions (Školáková et al., 2010) in the telomeric quadruplex support a noticeable tendency: it is not the lesion type but the position of the modification that determines the impact on the stability and conformation of the quadruplex. It has been found that most sensitive sites are located in the middle tetrad. Owing to the preference of oxoG for the *syn* conformation, distinct responses have been registered when guanines were replaced with various glycosidic conformations. OxoG accommodation at sites that were originally in an *anti* or *syn* conformation in a unsubstituted telomeric G-quadruplex requires, respectively, a slight structural rearrangement or a large conformational shift (BielskuteÌ et al., 2019). Nonetheless, the resulting structures still conform to a hybrid-type topology and are stable at physiological temperatures.

Despite the important functions that G-quadruplexes perform in gene transcription and telomere biology, the features of initiation of base excision processes by DNA glycosylases in non-canonical structures, including quadruplexes, are still poorly understood. In humans, 11 DNA glycosylases have been identified that specialize in the recognition and removal of various damaged bases. A comparison of the known structures of DNA glycosylases has uncovered six structural families having common structural domains: HhH-GPD, H2tH, UDG, AAG, ALK, and T4 Endo V (Brooks et al., 2013).

There are a few biochemical works (Zhou et al., 2013, 2015; Makasheva et al., 2020) showing how DNA glycosylases of two structural families H2tH (NEIL1, NEIL2, and mNeil3) and HhH-GPD (OGG1 and NTH1) remove oxidized lesions from a telomeric quadruplex or DNA bubble structures. It was shown that only mNeil3 exerts an excision activity on Tg in a quadruplex DNA. Nonetheless, guanidinohydantoin, and spiroiminodihydantoin, which are further oxidation products of oxoG, in quadruplex DNA, are good substrates for both mNeil3 and NEIL1 (Zhou et al., 2013). It was revealed that none of the glycosylases has an activity toward quadruplex DNA containing oxoG. Of note, a recently published (Brázda et al., 2018) detailed analysis of amino acid composition of all the known human G-quadruplex–binding proteins revealed a conserved RG-rich domain as a common feature of human G-quadruplex–binding proteins. Unexpectedly, this motif was identified in human enzymes NEIL1–NEIL3, which belong to the H2tH structural family.

To further evaluate the ability of DNA glycosylases that belong to different structural families to hydrolyze the *N*-glycosidic bond of damaged bases in G-quadruplexes and bulged structures, in this work, we performed a comparative analysis of the efficiency of damage removal by human DNA glycosylases NEIL1, OGG1, and NTH1. As a control, two prokaryotic enzymes from the H2tH structural family were examined: formamidopyrimidine-DNA glycosylase (Fpg) and endonuclease VIII (Nei), which are responsible for the removal of oxoG and Tg, respectively. Direct registration of product formation by polyacrylamide gel electrophoresis (PAGE) helped us to estimate the effectiveness of enzymatic hydrolysis of damaged G-quadruplexes under the same experimental conditions. The recognition of a specific site in the substrate is accompanied by a conformational adjustment (of the enzyme and DNA) optimizing specific contacts in the enzyme–substrate complex; therefore, we performed a kinetic pre–steady-state analysis of conformational changes in enzymes and damaged quadruplexes during their interaction. Conformational real-time rearrangements of DNA during the interaction with DNA glycosylases were visualized by detection of fluorescence reporters incorporated into DNA, e.g., the FAM/BHQ1 Förster resonance energy transfer (FRET) pair or a 2-aminopurine (aPu) residue on the 3’ or 5’ side of Tg or oxoG. aPu fluorescence intensity is sensitive to the microenvironment of this residue and enabled registering local conformational changes of a DNA substrate near the damaged nucleotide. On the other hand, the FAM/BHQ1-labeled damaged quadruplexes help to reveal “global” conformational changes that cause a change in the distance between the quencher and dye in the course of DNA substrate binding and cleavage.

## Materials and Methods

### Oligodeoxyribonucleotides

Sequences of the Oligodeoxyribonucleotides (ODNs) used in this study are listed in Table 1. The ODNs were synthesized by the standard phosphoramidite method on an ASM-700 synthesizer (BIOSSET, Novosibirsk, Russia) from phosphoramidites purchased from Glen Research (Sterling, VA, United States). Synthetic oligonucleotides were unloaded from the solid support with ammonium hydroxide, according to manufacturer protocols. Deprotected oligonucleotides were purified by high-performance liquid chromatography. Concentrations of the ODNs were calculated from their absorbance at 260 nm. ODN duplexes were prepared by annealing modified and complementary strands at a 1:1 molar ratio.

**TABLE 1:** Sequences of the ODNs, and structure of the modified residue^*a*^.

Name	Sequence	Description
Q4	5′-TTAGGGTTAGGGTTAGGGTTAGGGTT-3′	Control undamaged G-quadruplex
Q4-Tg	5′-FAM-TTAGGGTTAGGGT(Tg)AGGGTTAGGGTT-BHQ1-3′	G-quadruplex with Tg at 14th position (loop region)
Q4-aPu-Tg	5′-TTAGGGTTAGGG(aPu)(Tg)AGGGTTAGGGTT-3′	G-quadruplex with Tg at 14th position and aPu located on 3′ or 5′ side of damage
Q4-Tg-aPu	5′-TTAGGGTTAGGGT(Tg)(aPu)GGGTTAGGGTT-3′	
Q4-oxoG	5′-FAM-TTAGGGTTAGGGTTAG(oxoG)GTTAGGGTT-BHQ1-3′	G-quadruplex with oxoG at 17th position (in middle of GGG)
Q4-aPu-oxoG	5′-TTAGGGTTAGGGTTA(aPu)(oxoG)GTTAGGGTT-3′	G-quadruplex with oxoG at 17th position and aPu located on 3′ or 5′ side of damage
Q4-oxoG-aPu	5′-TTAGGGTTAGGGTTAG(oxoG)(aPu)TTAGGGTT-3′	
Type I Tg/A	5′-FAM- GCTCA(Tg)GTACAGAGCTG-3′	Control duplex with Tg; FAM and BHQ1 are located on one side of duplex
	3′-BHQ1-CGAGT A CATGTCTCGAC-5′	
Type II Tg/A	5′-FAM-GCTCA(Tg)GTACAGAGCTG-3′	Control duplex with Tg; FAM and BHQ1 are located on opposite sides of duplex
	3′- CGAGT A CATGTCTCGAC-BHQ1-5′	
Type I oxoG/C	5′-FAM- GCTCA(oxoG)GTACAGAGCTG-3′	Control duplex with oxoG; FAM and BHQ1 are located on one side of duplex
	3′-BHQ1-CGAGT C CATGTCTCGAC-5′	
Type II oxoG/C	5′-FAM-GCTCA(oxoG)GTACAGAGCTG-3′	Control duplex with oxoG; FAM and BHQ1 are located on opposite sides of duplex
	3′- CGAGT C CATGTCTCGAC-BHQ1-5′	
C/G	5′-GCTCACGTACAGAGCTG-3′	Control duplex without damage
	3′-CGAGTGCATGTCTCGAC-FAM-5′	
F/G	5′-GCTCA(F)GTACAGAGCTG-3′	Control duplex containing tetrahydrofuran (F) residue resembling abasic site, uncleavable by DNA glycosylases
	3′-CGAGT G CATGTCTCGAC-FAM-5′	
X/G	5′-FAM-GCGCATACGGCAT**X**ATCAGGGAAGTGGG-BHQ1-3′	Duplexes, X = oxoG or Tg
	3′- CGCGTATGCCGTA**G**TAGTCCCTTCACCC-5′	
X/-Δ1	5′-FAM-GCGCATACGGCAT**X**ATCAGGGAAGTGGG-BHQ1-3′	Bulge in damaged strand, X = oxoG or Tg
	3′- CGCGTATGCCGTA-TAGTCCCTTCACCC-5′	
X/-Δ2(5′)	5′-FAM-GCGCATACGGCAT**X**ATCAGGGAAGTGGG-BHQ1-3′	
	3′- CGCGTATGCCGT–TAGTCCCTTCACCC-5′	
X/-Δ2(3′)	5′-FAM-GCGCATACGGCAT**X**ATCAGGGAAGTGGG-BHQ1-3′	
	3′- CGCGTATGCCGTA–AGTCCCTTCACCC-5′	
X/-Δ3	5′-FAM-GCGCATACGGCAT**X**ATCAGGGAAGTGGG-BHQ1-3′	
	3′- CGCGTATGCCGT—AGTCCCTTCACCC-5′	
X/-Δ5	5′-FAM-GCGCATACGGCAT**X**ATCAGGGAAGTGGG-BHQ1-3′	
	3′- CGCGTATGCCG—–GTCCCTTCACCC-5′	
X/+Δ3	5′-FAM-GCGCATACGGCAT**-X-**ATCAGGGAAGTGGG-BHQ1-3′	Bulge in undamaged strand, X = oxoG or Tg
	3′- CGCGTATGCCGTA**GGG**TAGTCCCTTCACCC-5′	
X/+Δ4(5′)	5′-FAM-GCGCATACGGCAT**-X**–ATCAGGGAAGTGGG-BHQ1-3′	
	3′- CGCGTATGCCGTA**GGGC**TAGTCCCTTCACCC-5′	
X/+Δ4(3′)	5′-FAM-GCGCATACGGCAT**-X**–ATCAGGGAAGTGGG-BHQ1-3′	
	3′- CGCGTATGCCGTA**CGGG**TAGTCCCTTCACCC-5′	
X/+Δ5	5′-FAM-GCGCATACGGCAT–X–ATCAGGGAAGTGGG-BHQ1-3′	
	3′- CGCGTATGCCGTA**CGGGC**TAGTCCCTTCACCC-5′	
X/+Δ7	5′-FAM-GCGCATACGGCAT—X—ATCAGGGAAGTGGG-BHQ1-3′	
	3′- CGCGTATGCCGTA**CCGGGCC**TAGTCCCTTCACCC-5′	
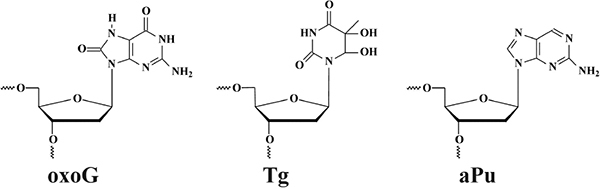		

*^*a*^aPu is fluorescent base 2-aminopurine, BHQ1 is black hole quencher, FAM is 6-carboxyfluorescein, oxoG is 8-oxoguanine, and Tg is thymine glycol.*

### Circular Dichroism Analysis

G-quadruplex folding was characterized by Circular dichroism (CD) spectroscopy using a JASCO spectrometer. The G-quadruplex samples were annealed at a 10 μM concentration in a buffer (50 mM Tris–HCl pH 7.5, 140 or 50 mM KCl, 1 mM EDTA) via heating of the samples at 90°C for 5 min followed by slow cooling down to room temperature. For each annealed G-quadruplex, CD analysis of each sample was performed by averaging of 16 CD scans at 25°C. Representative spectra of quadruplexes are shown in figures.

### Enzyme Purification

The human OGG1 protein was purified as described previously (Kuznetsov et al., 2014). The purification of full-length human endonuclease VIII-like NEIL1 was performed as reported before (Katafuchi et al., 2004). Formamidopyrimidine-DNA glycosylase (Fpg) and endonuclease VIII (Nei) from *Escherichia coli* were purified in their native form without tags or other modifications according to (Kuznetsova et al., 2019) and (Kladova et al., 2019), respectively.

Wild-type human endonuclease III NTH1 was isolated from *E. coli* Rosetta 2 cells transformed with plasmid pET28c-NTH1. Cells of *E. coli* Rosetta 2 were grown in the Luria–Bertani (LB) medium (1 L) containing 50 μg/mL kanamycin at 37°C to an optical density of 0.6–0.7 at 600 nm. After that, the temperature was lowered to 20°C, and transcription was induced by the addition of isopropyl-β-D-thiogalactopyranoside to 0.2 mM. Next, the cells were incubated for 16 h and then centrifuged (10,000 × *g*, 10 min). A cell suspension was prepared in 30 mL of buffer I (20 mM HEPES-NaOH pH 7.8) containing 40 mM NaCl and a protease inhibitor cocktail (Complete, Germany). The cells were lysed by means of a Thermo French Pressure Cell Press. All the subsequent procedures were conducted at 4°C. The cell lysate was centrifuged (40,000 × *g*, 40 min), and NaCl in the supernatant was adjusted to 200 mM; then the supernatant loaded onto column I (Q-Sepharose Fast Flow, Amersham Biosciences, Sweden) with subsequent washing with buffer I (20 mM HEPES-NaOH pH 7.8) containing 200 mM NaCl. Fractions containing the protein were collected and loaded onto column II (HiTrap-Chelating^TM^, Amersham Biosciences, Sweden) in buffer II (20 mM HEPES-NaOH pH 7.8, 500 mM NaCl, and 20 mM imidazole). Chromatography was performed in buffer II with a linear gradient of imidazole 20→500 mM. The solution’s absorbance was detected at a wavelength of 280 nm. The protein purity was determined by gel electrophoresis. Fractions containing the NTH1 protein were dialyzed against a buffer (20 mM HEPES-NaOH pH 7.5, 1 mM EDÒÀ, 1 mM dithiothreitol, 250 mM NaCl, and 50% of glycerol) and stored at –20°C.

Concentrations of all the enzymes were determined via the absorbance at 280 nm and an appropriate extinction coefficient. The activity of various proteins was tested by means of their classic substrates and was checked just before use.

### Microscale Thermophoresis Measurements

These measurements were carried out using Monolith NT.115 (NanoTemper Technologies). Monolith^TM^ NT.115 Standard Treated Capillaries were used. Each point on the fluorescence titration curves was obtained by measurement of the fluorescence intensity of separate solutions (10 μl) containing an oligonucleotide ligand (0.5 μM) and an enzyme at the required concentration in binding buffer (50 mM Tris–HCl pH 7.5, 50 mM KCl, 1 mM EDTA, 1 mM DTT, and 7% of glycerol). The mixtures were incubated at 25°C for 10 min. All titration experiments were repeated at least twice.

### Assays of Glycosylase Activity by PAGE

Single-turnover enzyme assays were performed in reaction buffer composed of 50 mM Tris–HCl pH 7.5, 50, or 140 mM KCl, 1.0 mM EDTA, 1.0 mM dithiothreitol, and 7% of glycerol (v/v). The reaction solution contained 2.0 μM enzyme and 1.0 μM DNA substrate. The reactions with damaged quadruplexes and duplexes were carried out at 25°C for 1 h or 5 min, respectively, and quenched by a gel-loading dye containing 7 M urea and 50 mM EDTA. Then, the reaction solutions were loaded on a 20% (w/v) polyacrylamide gel containing 7 M urea. The reactions with bulged substrates were carried out at 25°C for 5 min (Fpg and Nei) or 30 min (OGG1, NEIL1, and NTH1). The electrophoresis was run in 1 × TBE at 50 V/cm and 55°C. The gel was visualized using an E-Box CX.5 TS gel documentation system (Vilber Lourman, France). All the experiments were repeated at least twice. The degree of cleavage was calculated as the ratio of the peak area of the cleavage product to the sum of the peak areas of the product and the initial substrate. Error in the evaluation of the degree of cleavage did not exceed 20% usually.

### Stopped-Flow Experiments

We employed a SX20 stopped-flow spectrometer (Applied Photophysics Ltd., United Kingdom) equipped with a 150 W Xe arc lamp and an optical cell with 2 mm path length. The dead time of the instrument is 1.4 ms. The fluorescence of aPu was excited at λ_*ex*_ = 310 nm and monitored at λ_*em*_ > 370 nm as transmitted by filter WG-370 (Schott, Mainz, Germany). Fluorescence of a 6-carboxyfluorescein (FAM) residue was excited at λ_*ex*_ = 494 nm and monitored at λ_*em*_ > 515 nm as transmitted by filter OG-515 (Schott, Mainz, Germany). All experiments were carried out at 25°C in a buffer consisting of 50 mM Tris–HCl pH 7.5, 50, or 140 mM KCl, 1 mM EDTA, 1 mM DDT, and 7% of glycerol (v/v). The concentration of the enzyme in the reaction chamber after mixing in all experiments was 2.0 μM, and concentrations of a substrate were 1.0 μM. Typically, each trace shown is the average of four or more individual experiments; reported rate constants represent the mean (with standard deviation) of such datasets.

## Results and Discussion

### Structural Analysis of Quadruplexes Containing oxoG and Tg by CD Spectroscopy

**FIGURE 1:**
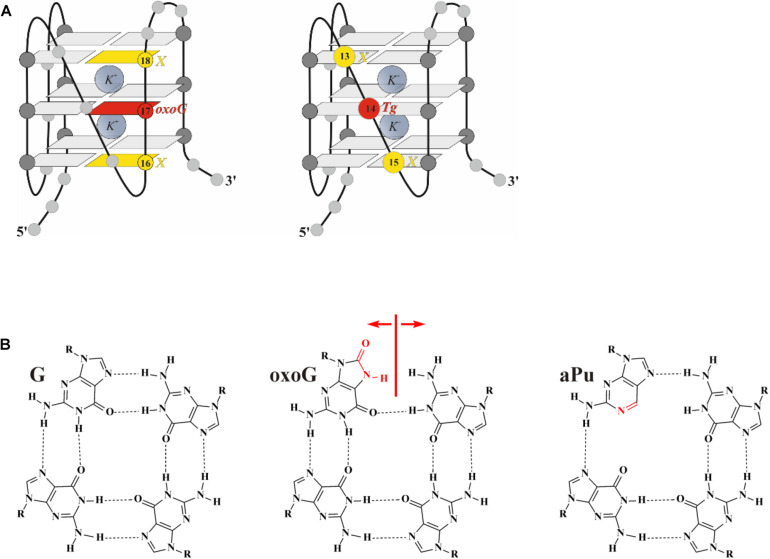
**(A)** The hybrid type of quadruplex folding. **(B)** Chemical structures of guanine (G), 2-aminopurine (aPu), and 8-oxoguanine (oxoG) in a G-quartet context. Positions 14 and 17, highlighted in red, were chosen to incorporate damaged nucleotide Tg and oxoG, respectively; positions 13, 15, 16, and 18, highlighted in yellow, were chosen to incorporate fluorescent nucleotide X.

Structural features of undamaged telomeric G-quadruplexes are well known (Parkinson et al., 2002; Ambrus et al., 2006; Phan et al., 2007; Burra et al., 2019). There are three conformational topologies, namely, a parallel, antiparallel, and hybrid fold. To obtain structural insights, we analyzed the ODNs under study by CD spectroscopy, under several buffering conditions. The ODNs were folded in a buffer containing 140 mM KCl, in which undamaged Q4 folds into a hybrid quadruplex structure (Figure 1A; Ambrus et al., 2006). Because high salt concentration can disrupt ionic enzyme–DNA contacts, we also utilized a buffer with 50 mM KCl, which is typically used for DNA glycosylase assays (Tchou et al., 1994; Jiang et al., 1997; Radicella et al., 1997; Hazra et al., 2002). Additionally, incorporation of Tg or oxoG as the damaged nucleotide in the loop region or at the core, respectively, can destabilize the quadruplex structure (Figure 1B). Moreover, the use of an aPu residue as a fluorescent reporter also leads to a loss of two hydrogen bonds in the G-tetrad.

**FIGURE 2:**
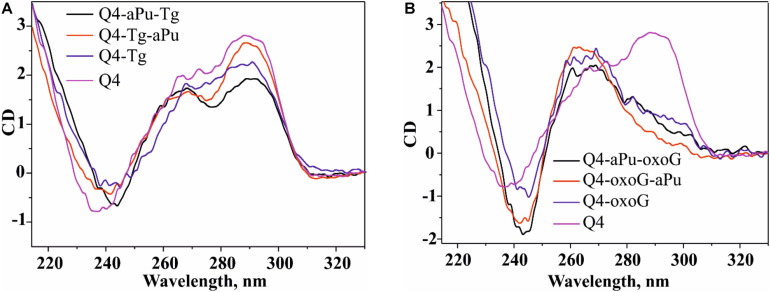
The CD spectra of the G-quadruplex containing Tg **(A)** or oxoG **(B)**. Concentration of G-quadruplexes was 10 μM. CD spectra were recorded at 25°C in the following buffer: 50 mM Tris–HCl pH 7.5, 140 mM KCl, 1 mM EDTA. Representative spectra of quadruplexes are given.

The CD spectrum of undamaged quadruplex Q4 in the buffer containing 140 mM KCl contains two pronounced maxima at 290 and 265 nm, supporting the hybrid-fold conformation (Figure 2A). Moreover, the CD spectrum profile of undamaged Q4 in the buffer with 50 mM KCl was very similar (data not shown), indicating that the changes of KCl concentration do not affect the G-quadruplex folding topology. Obtained CD spectra at both salt concentrations confirmed the hybrid-type structure, which appeared to be a stabler and thus predominant conformation of undamaged Q4 (Ambrus et al., 2006; Vorlíčková et al., 2012a).

It should be noted, that all quadruplexes containing Tg and aPu nucleotides in the loop region (Figure 1A, Q4-Tg, Q4-aPu-Tg, and Q4-Tg-aPu, respectively) yielded CD spectra similar to those of control undamaged Q4 (Figure 2A), indicating that these modifications of the loop region did not lead to a change of the hybrid-type folding.

In contrast, the ODN with oxoG in the middle position of GGG (Q4-oxoG) as well as ODNs additionally containing an aPu nucleotide on the 3’ or 5’ side of oxoG showed a drastic change in the CD spectra as compared with the native hybrid fold of undamaged Q4 (Figure 2B). Placement of the damage in the center of the GGG sequence gave a maximum peak at 263 nm and a minimum at 245 nm, which are typical of parallel G-quadruplex structures assumed by telomeric sequences under molecular-crowding conditions (Heddi and Phan, 2011). These data suggest that the oxoG modification of Q4 structure leads to obvious prevalence of a parallel conformation in the case of oxoG-containing substrates (Vorlíčková et al., 2005).

### Cleavage of Quadruplexes Containing oxoG and Tg

**FIGURE 3:**
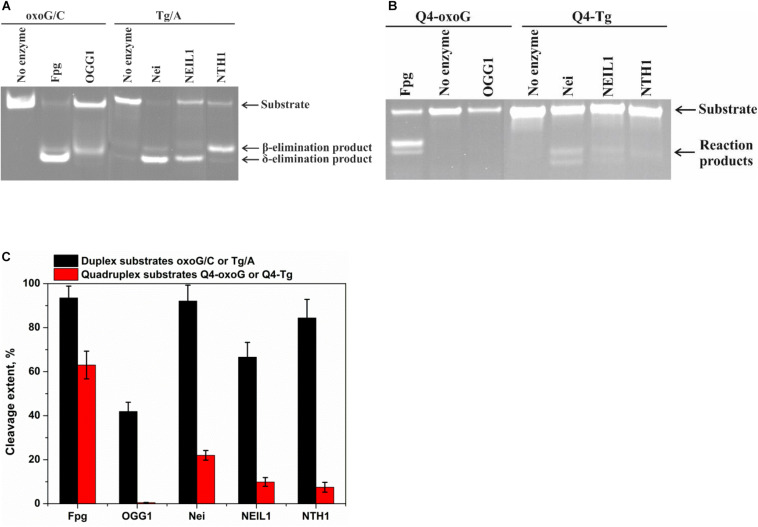
PAGE analysis of glycosylase activities toward the lesion-containing 17-nt DNA duplexes oxoG/C and Tg/A **(A)** and quadruplex DNA Q4-oxoG and Q4-Tg **(B)**. Comparison of the efficiency of cleavage of the tested substrates by DNA glycosylases **(C)**. [Enzyme] = 2 μM, [DNA] = 1 μM, Ò = 25°C, and reaction time = 5 min for duplexes or 1 h for quadruplexes. Experiments were repeated twice. Representative gels are shown.

The model random non-telomeric 17-nt duplexes containing specific damage, oxoG or Tg, were used to estimate the reference level of the glycosylase activity. As presented in Figure 3A, the cleavage of damaged nucleotides, oxoG or Tg, was performed by a specific DNA glycosylase. Nei, Fpg, and NEIL1 led to the formation of the reaction product corresponding to β,δ-elimination, whereas OGG1 and NTH1 generated the product corresponding only to β-elimination.

To measure the activity of DNA glycosylases toward quadruplex structures, Q4-oxoG, and Q4-Tg (Figure 3B) were used. The reaction mixture containing 2.0 μM enzyme and 1.0 μM DNA substrate was incubated at 25°C for 5 min or 1 h for duplex or quadruplex structures, respectively. The interaction of Fpg with Q4-oxoG led to the cleavage of the substrate at the oxoG position with ∼60% cleavage extent (Figure 3C). It should be noted that the interaction of OGG1 with Q4-oxoG did not cause substrate cleavage at all. The interaction of Nei with Q4-Tg resulted in the formation of both cleavage products corresponding to β-elimination and β,δ-elimination reactions with summarized efficiency of ∼25%. Nonetheless, the cleavage of Q4-Tg by NTH1 and NEIL1 was much less efficient and did not exceed 10% (Figure 3C).

### Binding of Quadruplexes Containing oxoG and Tg

**FIGURE 4:**
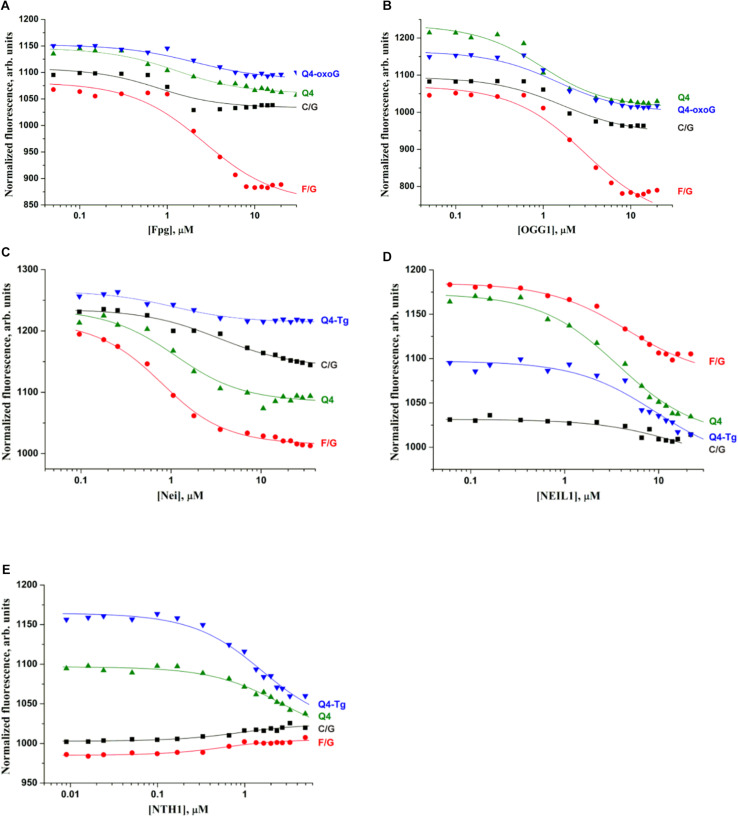
Titration curves characterizing the interaction of Fpg **(A)**, OGG1 **(B)**, Nei **(C)**, NEIL1 **(D)**, and NTH1 **(E)** with a native duplex without damage (C/G), a duplex containing an F-site (F/G), an undamaged G-quadruplex (Q4), and G-quadruplexes with Tg or oxoG (Q4-Tg or Q4-oxoG). Concentration of DNA was 0.5 μM. Titration was performed in the following buffer: 50 mM Tris–HCl pH 7.5, 50 mM KCl, 1 mM EDTA, 1 mM DTT, and 7% of glycerol.

Because low efficiency of cleavage of damaged quadruplexes can be associated with insufficient complex formation under the experimental conditions, we performed the Microscale thermophoresis (MST) assay to evaluate the binding constant corresponding to the interaction between DNA glycosylases and the quadruplex substrates. The MST technique allows to quickly measure the strength of the interaction between two molecules in solution by detecting a variation in the fluorescence signal of a fluorescently labeled DNA in a free state and in the complex with an enzyme. Therefore, the low efficiency of cleavage of quadruplex substrates during 1 h reaction time (Figure 3B) enabled us to carry out MST experiments without an influence of the DNA cleavage reaction. In the titration experiments (Figure 4), four types of DNA were tested: the control native duplex without damage (C/G), the control duplex containing an F-site (an uncleavable-by-DNA-glycosylases analog of an AP-site, F/G), the control G-quadruplex without damage (Q4), and G-quadruplexes with Tg or oxoG (Q4-Tg or Q4-oxoG). In some cases, the ratio of the signal to noise in the MST assay was not sufficient for precise calculation of dissociation constants *K*_*d*_. Therefore, all the obtained values were used only as estimates of the ability of enzymes to bind these structures. The obtained data are summarized in Table 2.

**TABLE 2:** The values of the dissociation constant *K*_*d*_ (μM) measured by MST.

Enzyme	Type of DNA				
	
	C/G	F/G	Q4	Q4-oxoG	Q4-Tg
Fpg	1.6 ± 1.1	1.9 ± 0.7	1.0 ± 0.3	1.2 ± 0.4	–
OGG1	1.4 ± 0.5	2.8 ± 0.7	0.7 ± 0.2	1.2 ± 0.3	–
Nei	3.1 ± 0.7	0.5 ± 0.06	0.9 ± 0.2	–	0.8 ± 0.2
NEIL1	17 ± 15	4.4 ± 0.9	3.4 ± 0.5	–	9.1 ± 3.0
NTH1	0.7 ± 0.4	0.3 ± 0.2	2.5 ± 0.8	–	1.3 ± 0.3

A comparison of dissociation constants *K*_*d*_ revealed good binding of DNA glycosylases to all the tested ODNs. Moreover, the values of constants were slightly dependent on both the structure of DNA and the presence of the lesion. These data indicate that the formation of even a non-specific complex between a DNA glycosylase and damaged DNA yielded sufficient changes of the signal in the MST assay. Nevertheless, the formation of such “primary” complexes was not enough to achieve a catalytic competent state with damaged quadruplexes. Indeed, similar DNA binding by different DNA glycosylases led to significantly different efficiency of cleavage of quadruplexes. Therefore, to further elucidate the features of DNA glycosylase interaction with Q4-DNA substrates, stopped-flow fluorescence measurements of real-time conformational transitions in substrates were conducted.

### Conformational Rearrangements in the Course of Interaction of OGG1 or Fpg With oxoG-Substrates Forming Quadruplexes

It has been previously shown by pre–steady-state analyses that the recognition of a damaged nucleotide in a duplex substrate is accompanied by a conformational adjustment of the damaged DNA; this process optimizes specific contacts in the enzyme–substrate complex in the case of either Fpg (Kuznetsov et al., 2007b, 2011, 2012) or OGG1 (Kuznetsov et al., 2007a, 2014; Kuznetsova et al., 2014; Lukina et al., 2017). The results of the kinetic analysis clearly indicated that Fpg and OGG1 can control substrate specificity via a multi-stage mechanism of recognition of a specific site, accompanied by conformational changes both in the DNA substrate and in the enzyme. The kinetic scheme of oxoG recognition includes a primary non-specific encounter, initial destabilization of the DNA around the damaged nucleotide, emergence of a kink in the DNA, eversion of the oxoG base from the duplex into the active site of the enzyme, filling of the consequent void in the DNA duplex, and finally, fine tuning of the enzyme and DNA resulting in a catalytically active conformation.

**FIGURE 5:**
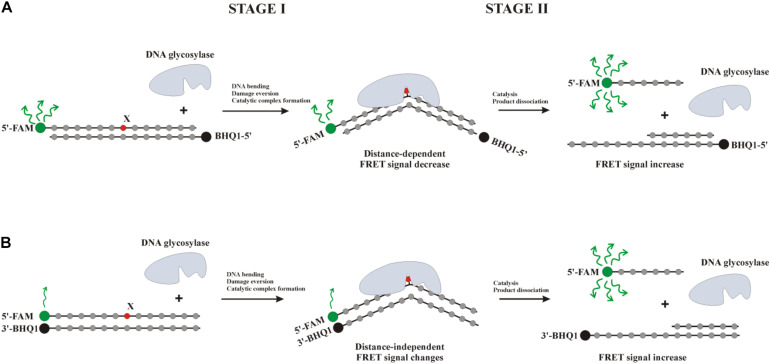
Interaction of DNA glycosylases with a type I **(A)** or type II **(B)** FRET-labeled substrate. Stage 1: the formation of an enzyme–substrate complex, accompanied by DNA duplex bending and damaged-nucleotide eversion; stage 2: elimination of the damaged base and cleavage of the AP-site, thus leading to the release of a short oligonucleotide fragment and an increase in the distance between FAM (fluorophore) and BHQ1 (quencher) accompanied by a significant increase in the FRET signal.

**FIGURE 6:**
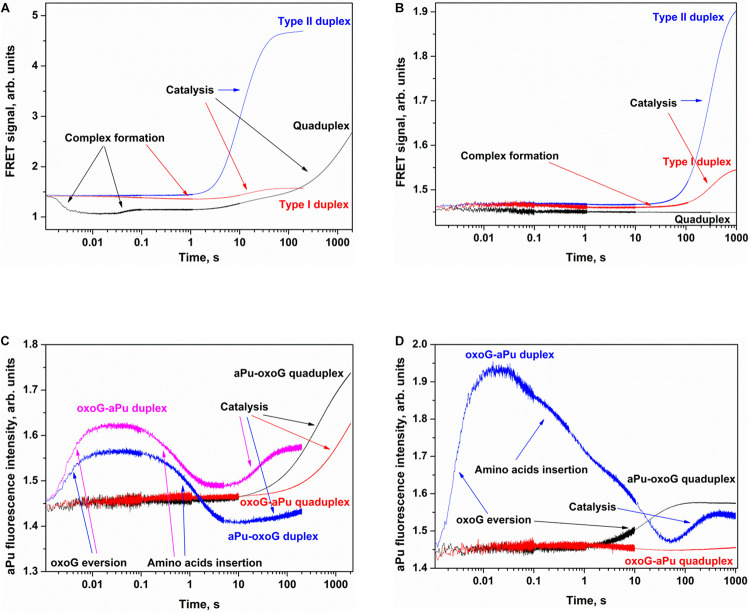
Processing of oxoG-containing duplexes and quadruplexes by Fpg **(A,C)** or OGG1 **(B,D)** as detected by means of changes in a FRET signal **(A,B)** or aPu fluorescence intensity **(C,D)**. Concentration of DNA substrates = 1 μM, and concentration of enzymes = 2 μM.

The kinetic pre–steady-state analysis of conformational changes of oxoG-containing quadruplexes in the course of interaction with OGG1 or Fpg was performed. Real-time conformational rearrangements of quadruplexes during the interactions with the enzymes were visualized via the detection of fluorescence of fluorescence reporters incorporated into DNA, e.g., the FAM/BHQ1 FRET pair or the aPu residue placed on the 3’ or 5’ side of oxoG. The FRET-labeled duplex substrates containing an oxoG lesion served as the control of substrate-binding and cleavage processes. For this purpose, two types of FRET-labeled duplexes were designed: type I duplex substrates (Figure 5A) contained FAM and BHQ1 at the 5’ ends of the ODNs; type II DNA substrates (Figure 5B) contained FAM at the 5’ end and BHQ1 at the 3’ end of the duplex-forming ODNs. The interaction of an enzyme with a model oxoG-containing DNA duplex (Figures 6A,B) led to DNA bending in the enzyme–substrate complex and thereby was accompanied by a decline in the FRET signal owing to shrinkage of the distance between FAM and quenching BHQ1 placed at the 5’ ends of the duplex-forming oligonucleotides (type I duplex). The emergence of the increase in the FRET signal at the end of kinetic traces for both types of duplex denotes the catalytic reaction and product release resulting in the growth of the distance between FAM and BHQ1. Moreover, expectedly, the type II DNA substrate containing FRET labels at the same end of the DNA duplex showed a greater change in the amplitude of the FRET signal.

The interaction of Fpg with type II oxoG/C led to strong growth of FAM fluorescence up to second 100 (Figure 6A), whereas the interaction of Fpg with type I oxoG/C duplex caused a FAM fluorescence decrease up to second 5 and then a FAM fluorescence increase up to second 100. The same FAM fluorescence changes were observed for the interaction of OGG1 with either type of oxoG/C duplex (Figure 6B). Therefore, the analysis of kinetic curves obtained for DNA duplexes allowed to clearly associate the changes of the FRET signal with different steps of the interaction of the enzymes with the substrate.

The interaction of Fpg with Q4-oxoG led to a fast initial decrease in FAM fluorescence with a subsequent increase up to millisecond 100 and a decrease up to second 2, indicating that the quadruplex-binding process is consistent with more complicated molecular events than the binding of duplex substrates. The slow two-phase increase in the FRET signal in the time range > 2 s denotes slow cleavage of the quadruplex and dissociation of the product (Figure 6A). On the other hand, the interaction of OGG1 with Q4-oxoG had no effect on FAM fluorescence (Figure 6B), suggesting that the binding of OGG1 to the quadruplex does not cause any changes in the distance between FAM and BHQ1.

Additionally, we used oxoG-containing duplexes and quadruplexes with aPu located on the 3′ or 5′ side of a damaged nucleotide (Figures 6C,D). The intensity of emission by aPu is strongly affected by its environment; for instance, it is quenched partially when aPu is stacked (Rachofsky et al., 2001). Detection of aPu fluorescence intensity in the course of the interaction of each enzyme with a duplex substrate revealed an increase of the intensity in the initial portion of the kinetic trace. According to previous reports on Fpg and OGG1 (Kuznetsov et al., 2007a,b; Kuznetsova et al., 2014), in this time interval, the oxoG residue flips out of the double helix, thereby leaving a void in the DNA helix. The subsequent introduction of amino acid residues of the active site into this void caused a subsequent decrease in aPu fluorescence intensity. The catalytic steps and dissociation of the enzyme–product complex caused the second increase in aPu fluorescence intensity owing to the transfer of this aPu to a more hydrophilic environment.

The interaction of Fpg with Q4-aPu-oxoG or Q4-oxoG-aPu did not lead to significant changes of fluorescence intensity in the initial portion of the kinetic curve. Nevertheless, at the end of the kinetic curve, an increase in aPu fluorescence intensity was observed (Figure 6C), pointing to a shift of aPu into more hydrophilic surroundings, possibly owing to destabilization of the quadruplex structure after the cleavage by the enzyme. These data suggest that oxoG eversion and catalytic-complex formation in the case of a quadruplex substrate is the rate-limiting step of DNA cleavage by Fpg. In this case, detection of the catalytic complex via aPu fluorescence failed due to an insufficient concentration of this complex in the reaction mixture. The interaction of OGG1 with Q4-oxoG-aPu had no effect on aPu fluorescence intensity (Figure 6D), supporting the notion that the enzyme cannot form appropriate contacts with the substrate and induce eversion of the oxoG base. Nevertheless, in the course of the interaction of OGG1 with Q4-aPu-oxoG, an increase in aPu fluorescence intensity was recorded, which suggests that the enzyme induces conformational changes in the quadruplex, and there are attempts of local melting and eversion of the damaged nucleotide into the enzyme active site.

Therefore, our findings imply that OGG1 cannot recognize the oxoG base located in the core of a quadruplex structure, whereas Fpg manages to form specific contacts with oxoG and to catalyze base removal and DNA cleavage.

### Kinetic Analysis of Interactions of Nei, NEIL1, or NTH1 With Each Tg-Containing Substrate Forming a Quadruplex

**FIGURE 7:**
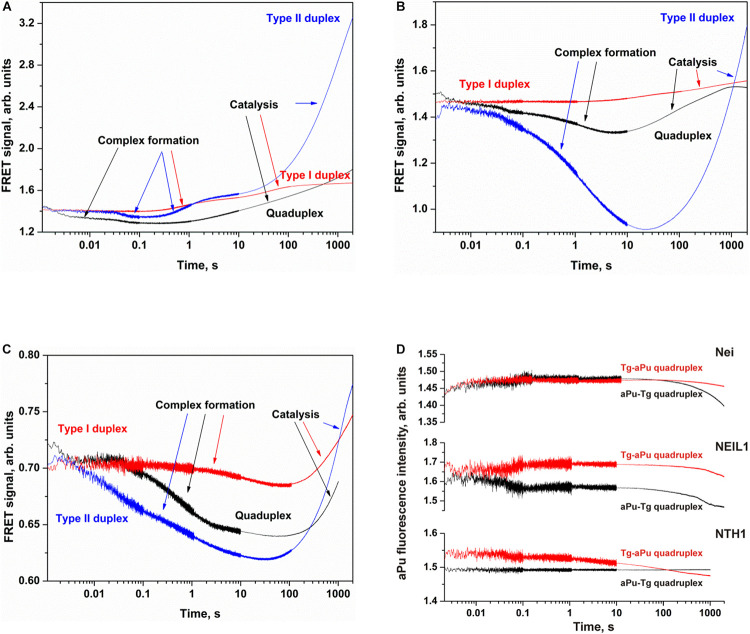
Processing of Tg-containing duplexes and quadruplexes by Nei **(A)**, NEIL1 **(B)**, and NTH1 **(C)** as detected by means of changes in a FRET signal or aPu fluorescence intensity **(D)**. Concentration of DNA substrates = 1 μM, and concentration of enzymes = 2 μM.

The same set of experiments was conducted with substrates containing Tg as a damaged nucleotide in the loop part of a quadruplex substrate. Two types of FRET-labeled Tg/A duplexes were used to assign the changes in the FRET signal to substrate-binding and catalysis steps. As depicted in Figure 7A, the interaction of Nei with type I Tg/A led to an increase in FAM fluorescence up to time point 300 s, whereas the interaction of Nei with type II Tg/A resulted in a fast FAM fluorescence decrease up to time point 0.1 s owing to the duplex bending and then to an increase of FAM fluorescence in the course of substrate cleavage. The same FAM fluorescence behavior was observed for the interaction of NEIL1 with either type of Tg/A duplex (Figure 7B). Of note, the interaction of NTH1 with these substrates caused a decrease in FAM fluorescence followed by an increase even in the case of the type I duplex substrate (Figure 7C).

The interaction of Nei or NEIL1 with Q4-Tg led to a decrease of FAM fluorescence in the initial part of kinetic curves (300 ms and 7 s for Nei and NEIL1, respectively), revealing the formation of an enzyme–substrate complex in which FAM/BHQ1 residues became closer to each other. The subsequent increase in the FRET signal indicates slow cleavage of the G-quadruplex (Figures 7A,B). The interaction of NTH1 with Q4-Tg led to an initial decrease phase of FAM fluorescence up to second 100, suggesting that the rate of substrate binding and damage recognition by NTH1 is much slower than that of Nei and NEIL1 (Figure 7C).

The interaction of Nei, NEIL1, or NTH1 with Q4-aPu-Tg or Q4-Tg-aPu did not cause significant changes in aPu fluorescence for all the enzymes (Figure 7D) regardless of the ability to be cleaved by these enzymes. These data uncovered low sensitivity of aPu fluorescence—when this residue is located in the single-stranded loop region of the quadruplex—to the processes of substrate binding and DNA cleavage.

### Cleavage of oxoG and Tg in the Bulged DNA Structures

**FIGURE 8:**
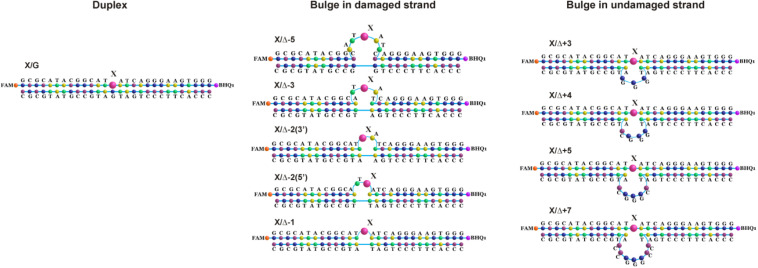
Schematic structures of X-containing DNA duplexes (X = oxoG or Tg) with bulging of a damaged (1–5 nucleotides) or undamaged (3–7 nucleotides) strand.

To further elucidate the mechanism of damaged nucleotide recognition in non–B-form DNA structures by DNA glycosylases, we designed a set of oxoG- and Tg-containing DNA duplexes with bulging of a damaged or undamaged strand having a bulge size of 1 to 5 or 3 to 7 nucleotides, respectively, (Figure 8). We analyzed by PAGE the efficacy of cleavage of a set of damaged DNA substrates that could facilitate DNA bending and the base eversion from the substrate into an enzyme’s active site.

**FIGURE 9:**
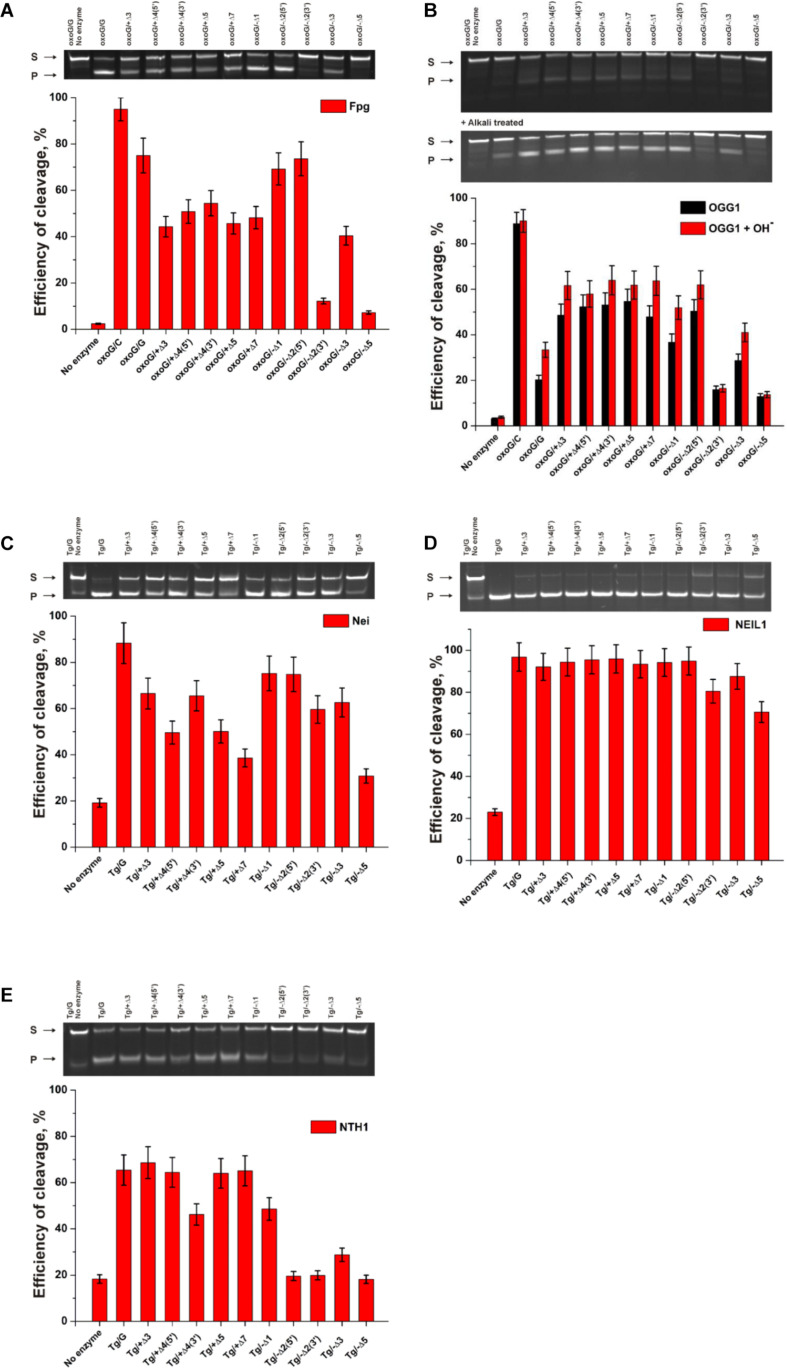
The efficiency of cleavage of oxoG-containing bulged DNA structures [by Fpg **(A)** or OGG1 **(B)**] and of Tg-containing bulged DNA structures by Nei **(C)**, NEIL1 **(D)**, and NTH1 **(E)**. [Enzyme] = 2 μM, [DNA] = 1 μM, Ò = 25°C, and reaction time = 5 min for Fpg, Nei, and NEIL1 or 30 min for OGG1 and NTH1. Experiments were repeated twice. Representative gels are shown.

The findings indicated that Fpg recognizes and removes oxoG from the oxoG/G pair with slightly lower efficiency as compared with the oxoG/C pair (Figure 9A, PAGE oxoG/C data are not shown). It is known that Fpg (Kuznetsov et al., 2007b) has opposite base specificity toward the oxoG/C pair as compared to oxoG/G, oxoG/T, and oxoG/A. It is noteworthy that the bulging of a single oxoG nucleotide (oxoG/-Δ1) or two nucleotides [oxoG/-Δ2(5’)] also slightly decreased the efficiency of DNA cleavage in comparison with the oxoG/C substrate. On the other hand, the strongest effect on the Fpg activity (Figure 9A) was observed in the case of bulging of 2 nucleotides in oxoG/-Δ2(3’), which contains an oxoG near the 3’ end of the duplex stem in the substrate, or 5 nucleotides (oxoG/-Δ5), which mimic single-stranded DNA. The bulge in the undamaged strand opposite oxoG reduced oxoG cleavage efficacy by approximately twofold regardless of the bulge size, suggesting that Fpg can place large nucleotide moieties outside the active site.

Of note, cleavage efficiency of OGG1 toward bulged DNA structures had features similar to those observed for Fpg (Figure 9B, PAGE oxoG/C data are not shown). Moreover, the two types of OGG1 activities, the cleavage of the *N*-glycosidic linkage and β-elimination, had similar profiles, indicating that recognition of oxoG limits the enzymatic activity. It was found that in the case of OGG1, just as the worst substrates—which contained the bulge of 2 [oxoG/-Δ2(3’)] or 5 nucleotides (oxoG/-Δ5)—the mismatched oxoG/G duplex was also a very poor substrate in comparison with oxoG/C. The strong preferential excision of oxoG from the oxoG/C pair in comparison with the oxoG/G pair is associated with stabilization of the cytosine base by many hydrogen bonds with Arg-154, Asn-149, and Arg-204 and van der Waals bonding to Tyr-203 (Bruner et al., 2000). The common mechanism of the damaged-nucleotide recognition by OGG1 was analyzed in detail elsewhere (Kuznetsova et al., 2014) and includes the formation of an initial transient enzyme–substrate complex in which sequential conformational changes of the enzyme and substrate are strongly needed for the formation of a catalytically active complex. The molecular processes that take place during these rearrangements include substrate bending, local melting of the duplex, damaged-nucleotide eversion from the substrate and insertion into the enzyme active site, amino acid residue insertion, and the formation of a network of contacts. There was high activity of OGG1 toward DNA substrates containing a bulge in the undamaged strand, just as in some cases of a bulge in a damaged strand [oxoG/-Δ1, oxoG/-Δ2(5’), and oxoG/-Δ3]. This observation suggests that facilitation of some of these processes (for example, the eversion of the damaged nucleotide or the bending of the substrate) may contribute the catalytic-complex formation because of the features of the initial structure of the duplex with the bulge.

A comparison of the efficiency of cleavage of Tg-containing duplexes by Nei (Figure 9C) and most clearly by NEIL1 (Figure 9D) uncovered a slight dependence on the location of the Tg nucleotide and on the size of the bulge. Indeed, in the case of Nei, the bulging in the undamaged or damaged strand decreased the enzymatic activity to ∼50% in comparison with a Tg/G duplex only in the cases of maximal bulge size (Tg/+Δ7 and Tg/−Δ5, respectively). At the same time, NEIL1 can efficiently cleave DNA regardless of the structure of the substrate. By contrast, NTH1 (Figure 9E), which belongs to the HhH structural family, manifested a significant decrease in cleavage efficiency toward structures with a 2–5-nucleotide bulge in the damaged strand but was mostly active toward structures with the bulge in the undamaged strand.

## Conclusion

One of the most important problems in studies on DNA glycosylases is elucidation of the enzymatic mechanisms that ensure precise recognition of a damaged base and its effective removal from DNA. Many DNA glycosylases have been structurally characterized to understand how damaged DNA bases are found and detected among numerous unmodified bases (Brooks et al., 2013). To solve this problem, we have performed pre–steady-state kinetic analyses of conformational changes in DNA glycosylases and in DNA substrates during their interactions (Kuznetsov and Fedorova, 2016, 2020).

DNA glycosylases of different structural families form completely different contacts in the substrate-binding site and in the active pocket, and different amino acid residues participate in specific recognition of the damaged nucleotide and in catalysis. Nonetheless, almost all DNA glycosylases have common features of interaction with substrates. Structural studies show that all DNA glycosylases bend the DNA molecule and flip out the damaged nucleotide from the DNA duplex. Moreover, as a rule, the damaged nucleotide is placed in the pocket of the active site, in which it is finally verified via formation of specific contacts with a damaged base, and some amino acid residues of the enzymes are inserted into the DNA void formed after base eversion. The sequence of these stages leads to the formation of the catalytic complex, in which the damaged nucleotide is optimally located in the active site of the enzyme for interactions with catalytic amino acid residues.

Our data indicate that the capacity for catalytic-complex formation with a non–B-form substrate depends on the structural family of the tested DNA glycosylases. Indeed, all these DNA glycosylases were able to form a complex with quadruplex DNA as revealed by MST, but pre–steady-state fluorescent analysis showed that OGG1 cannot form appropriate contacts with a quadruplex to induce oxoG eversion. Another enzyme of the same HhH structural family, NTH1, also has a barely noticeable activity toward a quadruplex containing Tg. On the other hand, all members of the H2tH structural family, i.e., Fpg, Nei, and NEIL1, can recognize a known cognate damaged nucleotide whether it is located in the loop or at the core of the quadruplex. From our findings about the bulged substrates, it can be concluded that DNA glycosylases OGG1 and NTH1 of the HhH structural family have much lower efficiency toward DNA substrates containing a bulge in the damaged strand. Nonetheless, some positions of a damaged nucleotide are poor substrates for members of both structural families, for example, the location near the 3’ end of the duplex stem in X/-Δ2(3’) substrates (X = oxoG or Tg), which contains a 2-nucleotide bulge, or the location in the 5-nucleotide bulge (X/-Δ5, X = oxoG or Tg), which mimics single-stranded DNA. It was found that NEIL1 can cleave Tg in all the tested bulged substrates as effectively as in the quadruplex. These results reveal that the biological function of this enzyme may be associated with the repair of non–B-form structures in DNA. Indeed, the ability of the tested enzymes to cleave damage-containing bulged DNA structures implies that these enzymes are important for the repair during transcription, replication, or recombination when single-stranded DNA and intermediate non–B-form structures, such as bubbles and bulges, can be formed. Nevertheless, our results suggest that the ability to cleave damaged quadruplexes is an intrinsic feature of members of the H2tH structural family, thus supporting the notion that these enzymes can participate in the modulation of processes controlled by the formation of quadruplex structures in genomic DNA.

## Data Availability Statement

The original contributions presented in the study are included in the article/supplementary material, further inquiries can be directed to the corresponding author/s.

## Author Contributions

AK conducted the experiments. NK conceived and designed the experiments. AK, NK, and OF analyzed the data. NK and OF contributed reagents, materials, and/or analytical tools. AK, NK, and OF wrote the manuscript. All authors contributed to the article and approved the submitted version.

## Conflict of Interest

The authors declare that the research was conducted in the absence of any commercial or financial relationships that could be construed as a potential conflict of interest.
